# Cellular Factors That Shape the Activity or Function of Nitric Oxide-Stimulated Soluble Guanylyl Cyclase

**DOI:** 10.3390/cells12030471

**Published:** 2023-02-01

**Authors:** Iraida Sharina, Emil Martin

**Affiliations:** Department of Internal Medicine, Cardiology Division, The University of Texas—McGovern Medical School, 1941 East Road, Houston, TX 77054, USA

**Keywords:** nitric oxide, cGMP, receptor, allosteric regulation, cell-derived factors

## Abstract

NO-stimulated guanylyl cyclase (SGC) is a hemoprotein that plays key roles in various physiological functions. SGC is a typical enzyme-linked receptor that combines the functions of a sensor for NO gas and cGMP generator. SGC possesses exclusive selectivity for NO and exhibits a very fast binding of NO, which allows it to function as a sensitive NO receptor. This review describes the effect of various cellular factors, such as additional NO, cell thiols, cell-derived small molecules and proteins on the function of SGC as cellular NO receptor. Due to its vital physiological function SGC is an important drug target. An increasing number of synthetic compounds that affect SGC activity via different mechanisms are discovered and brought to clinical trials and clinics. Cellular factors modifying the activity of SGC constitute an opportunity for improving the effectiveness of existing SGC-directed drugs and/or the creation of new therapeutic strategies.

## 1. Introduction

### 1.1. Cytosolic Guanylyl Cyclase Mediates Diverse Physiological Functions of NO

Nitric oxide (NO) is a gaseous diatomic molecule that acts as an intra- and extracellular messenger mediating diverse physiological and pathophysiological processes in various cells and tissues. NO exerts its function through two independent but overlapping pathways. One pathway relies on cGMP-dependent effector proteins, while another is cGMP-independent [1,2]. NO-stimulated guanylyl cyclase is an essential player in the NO/cGMP-signaling. This enzyme is often referred to as soluble guanylyl cyclase (SGC), due to its primarily cytosolic localization. Although multiple studies revealed that on many occasions, SGC is also found in membrane fractions of cell lysates and tissue homogenates [3,4], the term “SGC” remains most widely used. Soluble GC functions as a typical enzyme-linked receptor. Under resting conditions, SGC possesses weak cGMP-forming activity. However, following the binding of NO molecule to the SGC enzyme, cGMP-forming activity is activated several hundred-fold [5,6]. Elevated cellular cGMP level resulting from SGC activation engages cGMP-dependent kinases, phosphodiesterases, and cyclic nucleotide gated channels that affect a variety of cellular and physiological processes. These include calcium sequestration and cytoskeletal changes, relaxation of vascular smooth muscle cells (VSMC), improved oxygenation of tissues and organs [7], inhibition of adhesion and subsequent migration of leukocytes [8], reduction of platelet aggregation [9,10], facilitation of the repair of injured endothelium [11,12], inhibition of proliferation and migration of VSMCs [13], regulation of gastrointestinal motility [14], modulation of cancer development [15], and many others.

### 1.2. SGC Is a Highly Sensitive NO Receptor

SGC is a heterodimer which consists of one α and one β subunit. Humans and mice have two functional isoforms of the α subunit (α1 and α2) and one functional β_1_ isoform. The heterodimer α1β1 is ubiquitously expressed and has a higher level of expression. It has been recently classified as GC-1 [16]. The α2β1 heterodimer is classified as GC-2. GC-2 isoform is less abundant and is primarily expressed in the brain at the same level as the GC-1 counterpart. GC-2 is also detected in kidney and placenta. GC-1 and GC-2 are very similar in their structure and exhibit very similar responses to NO and other activating or inhibiting small molecules [17]. Nevertheless, there are some substantial differences in subcellular localization of GC-1 and GC-2. Ubiquitous GC-1 is mainly found in the cytosolic compartment of the cell, although a small fraction is membrane-associated [3]. In contrast, brain-expressed GC-2 isoform is primarily associated with the synaptic membrane [18], a property believed to be essential for neurotransmission [4,19].

**Figure 1:**
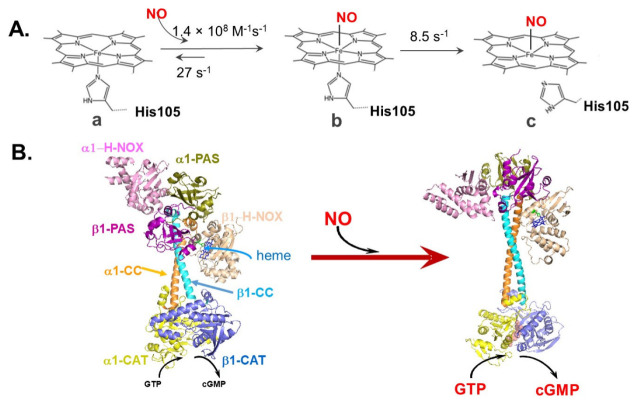
Structural changes of SGC in response to NO-dependent activation. (**A**): Schematic representation of the process of NO:-SGC adduct formation. The five-coordinate heme moiety of SGC (state a) binds NO to form a six-coordinate complex (state b). The six-coordinate complex subsequently converts irreversibly into a five-coordinate complex (state c) due to the rupture of Fe-His105 bond. Corresponding reaction rates are indicated. (**B**): Structural rearrangement of different SGC domains that occurs following NO binding causes significant activation of cGMP-forming activity.

The α and β subunits of SGC share a lot of sequence similarity and have similar domain organization. Each SGC subunit contains a heme nitric oxide/oxygen binding domain (H-NOX), a Per-Arnt-Sim domain (PAS), a coiled-coil domain (CC), and a catalytic domain (CAT). The β1 H-NOX domain harbors a heme prosthetic group [20], essential for binding the NO molecules. The structure of the α1β1 heterodimer established by cryogenic electron microscopy (cryo-EM) shows a two-lobe structure with the H-NOX/PAS domains on one connected by the CC domains end to the CAT domains on the other (Figure 1). Despite some differences between α1 and α2 sequences, the structure of the α2β1 isoform of SGC most likely follows the same fold.

Functional SGC is a hemoprotein containing a heme with ferrous iron. The heme moiety plays a key role in sensing the signal of elevated cellular NO levels. The heme is stabilized within the β1 H–NOX domain via coordination of the heme iron with His105 residue [6,21] and by interaction of the heme propionate groups with the Y135, Ser 137 and Arg 139 residues residing in the same domain [22,23]. The interaction of NO and SGC is a two-step process [24,25]. Initial binding of NO to the distal side of SGC heme results in the formation of a six-coordinate complex. However, unlike the stable complex between NO and heme in hemoglobin, the six-coordinate NO-heme complex of SGC is unstable. In a fraction of a second, the heme-His105 coordinate bond is disrupted (Figure 1A), maintaining only the NO-heme coordinate bond. The disruption of the His105-heme bond seems to release a conformational strain that triggers the relative rotation among α1 and β1 CC helices and straightening of the CC domains, while preserving some interactions between the β_1_ CC helix and the heme-containing β_1_ H-NOX domain (Figure 1B). The rotation of the CC helices causes a rotation of the catalytic CAT domains, resulting in changes in the GTP binding pocket [23,26]. These conformational changes only modestly lower the K_M_ for the GTP substrate [27], but significantly increase the V_max_ of the cGMP synthesis. V_max_ of the high-cGMP output state induced by NO is several hundred times higher than of the resting non-stimulated state [28,29].

Many hemeproteins with histidine as a proximal ligand for heme evolved to sense gaseous diatomic ligands. Although the major physiological function of different globins, such as hemoglobin, myoglobin, or cytoglobins, is to serve as sensors and carriers of molecular oxygen (O_2_), these proteins are also capable of binding carbon monoxide (CO) and NO with high affinity. Unlike these gaseous sensors, SGC evolved to have a unique ligand selectivity. Studies performed with purified SGC demonstrated that it cannot bind O_2_ even under high pressure of pure O_2_ [30]. It has been reported that exposure of purified SGC to saturating amounts of CO results in a modest 2–4-fold elevation of cGMP-forming activity [6,31]. However, careful examinations of the reaction between purified GC1 and CO revealed that GC-1 isoform exhibit a low affinity for CO, with an estimated K_D_ of 240–260 µM [30,32]. This value is more than four orders of magnitude higher than the estimated low nM level of physiological CO [33,34,35]. It seems unlikely that physiologically relevant CO-dependent activation of SGC takes place. The affinity of SGC for the NO ligand is much higher. Studies of interaction between purified GC-1 and NO solutions revealed a nanomolar affinity for NO (K_D_ 54 nM) [30,36,37]. Since both SGC isoforms exhibit similar dose-dependent increase of cGMP-forming activity in response to NO donors [38], it is highly probable that the affinity for NO is very similar. Thus, SGC has a strong selectivity towards NO as the main physiological activating agent, as it is expected from a NO receptor. While the gaseous ligand selectivity of GC-1 and GC-2 was not compared, it is reasonable to assume that both SGC isoforms have similar affinities for signaling gasses.

However, if the affinity for NO is regarded as the main parameter determining the role of a protein as a highly sensitive NO receptor, SGC does not seem to fit the role. A number of intracellular histidine ligated hemoproteins have higher affinities for NO than SGC [39]. Thus, it is important to consider physiological levels of NO and SGC. Direct measurements of NO produced in different cells [40,41] and the assessment of bioavailable NO suggest that physiological levels of NO reach subnanomolar concentrations [40,42]. The EC_50_ values for various NO donors sufficient to elicit a desired physiological response is often in the range of 100 pM to 5 nM [40]. These are much lower values than the ~50 nM K_D_ for NO determined *in vitro* with purified SGC. Thus, under normal physiological conditions, SGC encounters concentrations of NO much lower than its calculated K_D_ value. Yet, these NO concentrations are sufficient to generate a physiological response. Therefore, the K_D_ value determined at equilibrium and reflecting the affinity for NO is not an appropriate parameter to determine if SGC is an efficient NO receptor. Considering that the waves of NO generated by activated eNOS and nNOS are transient, these levels of NO are not sustained long to establish an equilibrium condition. Therefore, the NO binding constant is a parameter better suited to judge the effectiveness of SGC as NO receptor. While SGC’s affinity for NO is not the highest among known hemoproteins, the kinetics of NO binding to SGC heme is very fast. A number of studies directly measured the kinetics of NO interaction with SGC heme and determined the association constant k_on_ to be in the 1.4–4.5 × 10^8^ M^−1^s^−1^ range [24,29,30,43]. This is a diffusion-limited binding and the fastest NO binding among proteins known to interact with NO. Therefore, in a cellular environment containing many proteins competing for NO binding, the binding kinetics of NO to the ferrous SGC heme favors the formation of NO:SGC adduct and subsequent activation of cGMP-forming activity.

Once formed, the NO:SGC complex should be quite labile to be appropriate for various rapid signaling processes that depends on NO/cGMP signling. NO dissociation measured spectroscopically resulted in a half-life of the NO:SGC complex of approximately 2 min [44]. A similar half-life of approximately 3 min was reported in a different study [45]. These values obtained *in vitro* with purified SGC are not compatible with fast deactivation required for efficient NO signaling and observed experimentally. For example, studies performed on aortic rings demonstrated that relaxation of aortic rings can be re-elicited 1–2 min after previous exposure to NO [46]. This discrepancy most likely reflects the contribution of different cellular factors. *In vitro* spectroscopic studies demonstrated that some cellular factors may accelerate the process of NO:SGC decomposition. For example, in the presence of different thiols (DTT, GSH, cysteine) the half-life of the complex is much shorter than without thiols [45], while the addition of Mg^2+^-GTP yielded a half-life of 5 s [47]. Deactivation of NO:SGC determined by monitoring the decline in cGMP-forming activity yielded a similar ~5 s value for purified protein [48] and cytosolic fraction of bovine retina [49]. Even faster deactivation was reported in case of intact cerebellar cells, where the estimated half-life was 0.2 s [50].

Equally important for SGC function as an efficient NO receptor is the abundance of SGC protein in physiological systems responsive to NO. It has been estimated that intracellular concentrations of SGC, at least in platelets and cerebellar astrocytes, reaches micromolar range [51]. In mouse aorta, the amount of SGC far exceeds the amount needed to mediate the relaxation of aortic smooth muscles. The loss of functional GC-1 in mice lacking α1 SGC subunits was functionally compensated by GC-2 [52], which constitutes only 6% of the total SGC activity in aorta. The large excess of SGC over the bioavailable NO coupled with the fast-binding kinetics ensures that a sufficient number of SGC molecules is activated to achieve the desired physiological outcome.

*In vitro* studies with purified SGC heterodimer clearly demonstrate that the presence of Mg^2+^-GTP and NO is sufficient to promote NO-dependent activation of cGMP-forming activity. However, multiple data indicate that there is a number of cellular factors affecting either positively or negatively this process. For example, SGC has a higher sensitivity for NO [51,53] in intact cells than *in vitro*. The rate of NO dissociation from SGC heme in cerebellar cells is 25 times higher than the one observed *in vitro* with purified protein [50]. There is multiple evidence of SGC desensitization in vivo, but no desensitization is observed with the purified enzyme *in vitro* [54]. A number of studies reported that some cells contain factors affecting SGC activity. For example, the lysates of endothelial cells contain a heat-labile activator of SGC [55], while COS-7 cells contain factor(s) that strongly enhance the activity of resting and NO-activated purified SGC [56]. Studies of the last two and half decades demonstrated that SGC activity may be upregulated via allosteric modulation by synthetic small molecules. Two types of such allosteric regulators have been identified [57], namely SGC stimulators and SGC activators. Allosteric stimulators of SGC strongly potentiate NO signaling by sensitizing the enzyme to low doses of NO [57]. Many of these stimulators are undergoing clinical trials at different stages. At least two stimulators, riociguat and vericiguat, were approved as SGC-targeting therapeutics for the management of pulmonary arterial hypertension, chronic thromboembolic pulmonary hypertension [58,59], and heart failure conditions [60,61]. Allosteric activators seem to target the β1 H-NOX domain and activate NO-independently the enzyme that lacks heme or contains oxidized ferric heme [62]. They are also promising drug candidates [63]. The existence of such synthetic allosteric regulators suggests the potential existence of a cellular factor(s) that affect(s) the activity of SGC in a similar fashion. This manuscript reviews the effect of cellular factors known to directly modulate the function of SGC as a cellular NO receptor. Factors affecting the expression of SGC subunits or influencing SGC function indirectly are not discussed.

## 2. Modulation of SGC Activity by Cell- and Tissue-Derived Small Molecules

### 2.1. Role of Additional NO as Regulating Cellular Factor

In a 2004 study, Russwurm and Koelsing investigated the relationship between the extent of SGC activation and the amount of NO donors present in reaction mixture. They reported that exposing SGC to an equimolar amount of NO is sufficient to generate the NO:SGC adduct, according to the spectral evidence. However, such NO:SGC adduct does not exhibit maximal cGMP-forming activity [64]. They observed that only by providing additional NO the maximal activity can be achieved, suggesting that more than one NO molecule is required for full activation. Later studies by other groups confirmed these observations [36,65]. In the context of these findings, NO may be regarded not only as an activating heme ligand, but also as an allosteric cellular factor. It should be noted that the study by Russwurm and Koesling and several later studies established that when the NO:SGC adduct is formed in the presence of GTP, stoichiometric amount of NO is sufficient to achieve maximal stimulation of SGC without the need for additional NO [36,64,65].

**Figure 2:**
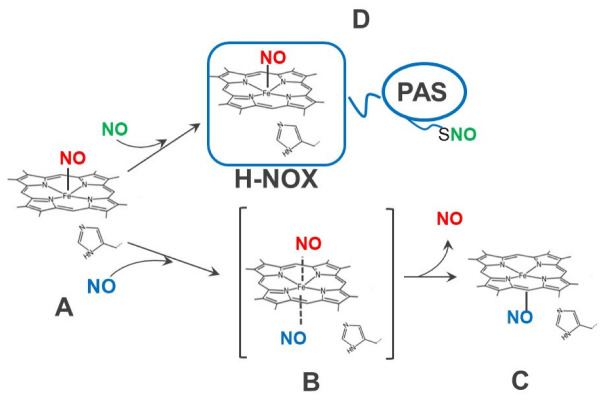
Alternative hypothesis of the allosteric effect of additional NO. In the presence of NO excess, the five-coordinate NO-heme adduct (state **A**) may bind a second NO to form a transient ternary complex (state **B**), which rapidly loses the distal NO and converts into a five-coordinate NO-heme adduct with NO bound on the proximal side of heme (state **C**). Alternatively, the allosteric effect of additional NO is explained by S-nitrosylation of an unspecified cysteine residue (state **D**), resulting in a full stimulation cGMP-forming activity.

By applying carefully timed sequential addition of ^14^NO and ^15^NO ligands with subsequent rapid freezing at different time points and EPR analysis, it was demonstrated that the second NO molecule binds to the proximal side of SGC heme (Figure 2, state C) [66]. Similar NO-heme adduct with NO on the proximal side was observed in the X-ray structure of the NO-bound state of cytochrome c’ of the bacterium *Alcaligenes xylosoxidans* [67]. Later studies of the NO-bound H-NOX protein from *Shewanella oneidensis* (So H-NOX) [68] also reported a similar location of the NO ligand. It has been proposed that the formation of such NO-heme adduct with NO on the proximal side may stabilize the activated state of SGC and explains how maximal cGMP activity is achieved [36,37,66,69].

Studies by another group proposed an alternative mechanism that explains the stimulatory effect of the additional NO molecule. The authors demonstrated that SGC pretreated with methyl methanethiosulfonate (MMTS), a thiol reactive compound, does not exhibit full activation, even when excess NO donor was applied. Thus, a putative protein modification of SGC in the form of nitrosothiol or thionitroxide has been postulated [70] (Figure 2, state D), but the specific cysteine residues involved remains to be determined.

Regardless of the mechanism by which additional NO exerts its stimulatory action on purified SGC, it is not clear if this effect is reproduced in the cellular environment. As already mentioned above, the stimulatory effect of additional NO is not needed to achieve maximal activity when the reaction happens in the presence of GTP [36,65]. Considering that intracellular concentrations of GTP is estimated to be around 100–200 µM [71,72] and the lower K_M_(GTP) value (~70 µM ) for purified SGC [73], most of the intracellular SGC should be in the GTP bound form. Another important factor to be considered is the physiological ratio of produced NO to available SGC enzyme. As already mentioned, the physiological level of NO in different cells reaches subnanomolar concentrations [40,41,42], while SGC level may reach micromolar levels. Thus, under normal physiological conditions, the cells exhibiting a robust NO/cGMP-dependent signaling have a substantial excess of the NO receptor over the bioavailable NO, making it unlikely for NO to have an allosteric function. Therefore, the allosteric role of NO most likely may exhibit itself only in inflammatory conditions, when there is a sustained production of NO due to the activity of inducible nitric oxide synthase (iNOS).

The synthesis of NO under conditions of oxidative stress results in byprodact formation of nitrosylated thiols of various nature [74], including free-thiol cysteines in proteins. Early studies by Ignarro and colleagues implicated nitrosothiols in activation of SGC in different cell models [75,76,77]. These observations reflect the instability of different nitrosothiols, which are susceptible to reduction and release of captured NO. Several decades later, the studies by Beuve and colleagues demonstrated that the results are quite different if the decomposition of nitrosothiols is being carefully controlled. They demonstrated that following the pretreatment with S-nitrosating agent S-nitrosocysteine (CSNO) SGC exhibits a significant decrease in responsiveness to NO donors [78], essentially demonstrating desensitization of SGC. In a series of studies, Beuve and colleagues demonstrated S-nitrosylation of SGC in various systems and conditions. They reported S-nitrosylation of SGC in human umbilical vein endothelial cells treated with vascular endothelial growth factor; in isolated aorta after prolonged exposure to acetylcholine [78]; in aortic smooth muscle cells exposed to nitrovasodilator nitroglycerin [79]; in aldosterone-treated bovine vascular smooth muscle cells [80]; in a hypertensive model of angiotensin II-treated rats [81]. Nitrosative desensitization of SGC was also confirmed with different S-nitrosating agents [82] or in mice overexpressing endothelial NOS [83]. Initial studies revealed that two cysteine residues in the α1 and β1 H-NOX domain, αC243 and βC122, are responsible for SGC desensitization via nitrosylation [78]. However, subsequent thorough proteomic examination revealed additional SGC cysteine residues that may be susceptible to S-nitrosylation and contribute to desensitization [78,79,81,83,84,85]. In summary, many studies clearly demonstrate that NO plays not only the role of heme ligand that activates cGMP-forming activity of SGC, but also may act as a modulator of SGC activity and function in specific conditions.

### 2.2. Role of Free Cellular Thiols in SGC Function

A simple analysis of the amino acid composition of human SGC reveals a high content of Cys residues. Using a large protein data set as reference, Marino and Gladyshev estimated that the frequency of cysteine residues in an average cytosolic protein is ~1.59% [86]. However, the frequency of cysteine residues in the α1 and β1 subunits is 3.3% and 2.3%, respectively. As described above, many of these Cys residues may be nitrosylated, affecting the extent of SGC activation by NO. However, other processes involving Cys residues, such as disulfide bond formation, oxidation to sulfenic acid, or sulfonation may take place. Early studies of SGC demonstrated the susceptibility of the enzyme to different thiol-targeting agents. For example, SGC activity in hepatic cells was inhibited by arsenite, which interacts with cysteines, but reversed by the thiol reducing agent dimercaprol [87]. Purified enzyme was inhibited by agents inducing disulfide bonds, but the inhibition was restored by thiol-reducing dithiothreitol (DTT) [88,89]. One study demonstrated that ^35^S-labeled cystin was incorporated into the purified SGC and this label was released by DTT [88]. Collectively, these studies indicate that a number of Cys residues of SGC may be susceptible to disulfide bond formation or other type of cysteine oxidation/modification, which may affect the NO receptor function of SGC. In recent years, a series of reports using proteomic and bioinformatic tools identified a number of SGC cysteine residues that may be involved in these processes [85,90,91].

Since most of the inhibitory effects of cysteine-targeting agents were reversed by different thiols, the early studies uncovered the importance of cellular thiols in maintaining SGC function. Early studies on biochemical purification of SGC noticed a loss of NO responsiveness of SGC during purification, which can be abolished by the addition of DTT [92]. More recently, it has been demonstrated that cellular thiols, such as GSH, play an important role in modulating the effect of SGC cysteine nitrosylation. It has been shown that incubation with GSH reverses the desensitization of SGC caused by cysteine nitrosylation and at certain doses prevents the nitrosylation from taking place [82].

Thiols were also shown to play an important role in maintaining the proper redox state of the heme moiety in SGC. For NO signal recognition, it is important that SGC heme iron is in the reduced ferrous state. Oxidation of SGC heme prevents the binding of NO and activation of SGC. Moreover, SGC with oxidized heme has the tendency to lose heme [93] and is more susceptible to degradation [94,95,96]. The discovery of NO-independent activators of SGC allowed a better understanding of the widespread occurrence of SGC with oxidized heme or even lacking heme. As mentioned above, allosteric activators of SGC activate SGC more efficiently when the heme moiety is lacking or it is in the oxidized state [62]. Using these agents as probing tools, multiple studies demonstrated that even under normal physiological conditions, these activators cause a substantial increase in cellular and tissue cGMP [94,97,98,99,100]. These studies support the notion that there is a substantial portion of cellular and tissue SGC with oxidized heme. The biochemical properties of a rare mutant variant of human SGC associated with hypertension, achalasia, and moyamoya conditions [101] was recently reported. The Cys517→Tyr substitution in the α1 subunit rendered the mutant SGC heme more susceptible to oxidation, causing a higher rate of degradation of the mutant SGC under oxidative condition [27]. Previous studies demonstrated that some thiols, such as DTT, are effective reducing agents that cause the reduction of oxidized SGC heme [102]. After exposure to DTT, SGC with oxidized heme fully restored its ability to bind NO and activate cGMP-forming activity. In the case of mutant variant α_1_C517Yβ_1_, such protective effect of cellular thiols is impaired, underlying the observed pathological phenotype [27]. While the mechanism of such thiol-mediated reduction of SGC heme remains to be understood, it underscores the importance of cellular thiols as cellular factors modulating and supporting the function of SGC.

Hydrogen sulfide is another cellular factor affecting the redox state of SGC heme. Three enzymes are recognized as endogenous sources of H_2_S in various cells and tissues: cystathionine γ-lyase (CSE), cystathionine β-synthase (CBS), and 3-mercaptopyruvate sulfurtransferase [103]. When SGC with ferric heme was exposed to hydrogen sulfide, the heme was reduced to ferrous form, coinciding with restored ability to bind NO and stimulate cGMP-forming activity [104]. Studies in cultured rat aortic smooth muscle cells and mouse aortic rings provide supportive evidence that H_2_S-mediated reduction of ferric SGC heme is a physiological process that facilitates NO-mediated cellular signaling [104].

### 2.3. Role of Ca^2+^ Ion in the Activity of SGC

Increased intracellular Ca^2+^ is essential for activation of endothelial and neuronal nitric oxide synthases [105]. A number of studies also demonstrate that changes in intracellular Ca^2+^ directly affect the activity and function of SGC. Initial studies performed on purified SGC revealed that Ca^2+^ inhibits SGC [106,107]. Calcium treatment dramatically decreased V_max_ and K_M_(GTP) for purified SGC or SGC expressed in different cell lines [107,108]. Mechanistic studies unmasked the negative allosteric sites with high (Ki~10 µM) and low (Ki~100 µM) affinities for Ca^2+^ mediating noncompetitive and uncompetitive inhibition, respectively [109]. The same study reported that purified SGC binds ^45^Ca^2+^ even in the presence of a large excess of Mg^2+^ ion, demonstrating that SGC is a constitutive Ca^2+^-binding protein. The nature of this Ca^2+^-binding site remains to be determined. Studies in different cell lines demonstrated that carbachol-, thrombospondin-1-, or angiotensin II-induced increase of cellular Ca^2+^ lowers SGC activity [107,108]. Depolarization of pituitary cells by high K^+^ and L-type Ca^2+^-channel agonists increased intracellular Ca^2+^ and blunted the elevation of cellular cGMP in response to NO [110]. Similar effect to elevated cellular Ca^2+^ was also reported in pancreatic acinar cells [111]. On the other hand, studies in cytosolic fraction of bovine retina revealed that Ca^2+^ slows down the deactivation of SGC [49].

Elevated Ca^2+^ not only affects the activity of SGC, but also influences its subcellular localization. It was reported that increased Ca^2+^ promotes translocation of SGC to the membrane fraction of human platelets and to the caveolar fraction of lung endothelial cell [3]. Interestingly, the membrane-associated SGC was more sensitive to lower doses of NO donor than cytosolic SGC. It remains to be determined if Ca^2+^-induced translocation of SGC to the membrane is dependent on the direct binding of Ca^2+^ to one of the Ca^2+^-binding sites on SGC or if this is a PKC-dependent process.

### 2.4. Cell- and Tissue-Derived Allosteric Factors

The discovery of synthetic allosteric regulators of SGC activity points to the possibility that various small molecules of cellular origin may possess identical or similar function and affect the cGMP-forming activity of SGC. Synthetic allosteric stimulators on their own activate SGC relatively weakly. However, in the presence of these stimulators, SGC exhibits a robust activation by low concentration of NO, which usually do not elicit any substantial effects [112]. The cryoEM studies demonstrated that the synthetic allosteric stimulator YC-1 binds directly between the beta H-NOX domain and the two CC domains [113]. Other studies revealed that cobinamide, a naturally occurring precursor of vitamin B12, shares some functional similarities with synthetic stimulators. Cobinamide weakly activates sGC, but significantly enhances the effect of YC-1, reaching levels of activation observed with moderate concentrations of NO donors [114]. No stimulation of NO-dependent activation was observed with cobinamide. The binding of cobinamide was also mapped to the fragment of SGC protein containing the CC and the catalytic region. Although relaxation of isolated aortic rings in response to cobinamide was demonstrated in organ baths, the physiological role of cobinamide-dependent activation of SGC remains to be demonstrated. Since symbiotic microbiome and diet are the main source of vitamin B12 in humans [115], it is possible that cobinamide-dependent regulation of SGC is restricted to the GI tract, where SGC plays an important role in motility [14].

As discussed above, the discovery of SGC allosteric activators that act by occupying the empty heme pocket [116] allowed the discovery that a substantial portion of SGC lacks heme. Long before the discovery of synthetic allosteric SGC activators, Ignarro’s research team reported that SGC is highly activated by protoporphyrin IX [117], a precursor in heme synthesis. Later studies demonstrated that protoporphyrin IX is an effective activator only of SGC lacking heme [118]. Therefore, protoporphyrin IX may be regarded as a potential cell-derived allosteric activator on SGC. Interestingly, the product of heme breakdown by heme oxygenase has an opposite effect. It has been demonstrated that biliverdin IX significantly decreases both basal and NO-stimulated activities of SGC [119], presumably by binding to the heme pocket and displacing the heme. Another potential SGC inhibiting small molecule synthesized by cells is carnosine. Carnosine, a dipeptide *beta*-alanyl-L-histidine synthesized in the liver [120], is known for its anti-oxidant properties [121]. Several studies reported that carnosine inhibits NO-dependent activation of SGC [122,123]. It has been speculated that carnosine may chelate the heme iron, thus interfering with the binding of NO [124].

## 3. Modulation of SGC Activity by Cellular Proteins

In addition to cell-derived small molecules that directly affect SGC, a growing list of proteins has been reported to shape SGC activity and function.

### 3.1. Proteins Affecting the Redox Status of SGC Thiols 

The high frequency of cysteine residues in SGC provides ample opportunities for misfolding in a thiol oxidation environment. Oxidoreductase protein disulfide isomerase (PDI) is an enzyme in the endoplasmic reticulum (ER) that catalyzes the formation and breakage of disulfide bonds between cysteine residues within proteins during the maturation process [125]. PDI was reported to interact with purified SGC and inhibit its activity [126]. This interaction appears to be via the formation of a mixed thiol-disulfide bond between SGC and PDI. The biological relevance of such interaction remains to be determined, considering that PDI is primarily in ER, while SGC is exclusively outside of ER. The cytosolic oxidoreductase thioredoxin-1 also associates with SGC via a similar mixed disulfide [127]. Thioredoxin-1 was shown to reverse the S-nitrosylation of SGC and protect the enzyme from desensitization. Considering the importance of cysteine residues of SGC, it is highly plausible that other cellular proteins affecting cellular thiol redox status may indirectly modulate the function of SGC.

### 3.2. Proteins Affecting the Redox Status of SGC Heme

Maintaining the heme moiety in a reduced state is essential for the binding of NO and the function of SGC as NO receptor. In 1999, Gupte and colleagues reported that a flavoprotein-containing NADPH oxidoreductase is important in restoring the sensitivity to NO for SGC with oxidized heme [128]. NADPH was essential for this restoration. In later studies, it was reported that Cytochrome B5 reductase (Cyb5R3) is important for maintaining the reduced state of SGC heme [98]. Cyb5R3 is also known as methemoglobin reductase and requires NADPH for its function. Mice lacking Cyb5R3 in vascular smooth muscle cells exhibit higher levels of SGC containing oxidized heme [100].

### 3.3. Proteins Affecting the Assembly of SGC Heterodimer 

The assembly of functional heme-containing SGC heterodimer is a stepwise process requiring the involvement of additional interacting proteins. Careful studies by the Stuehr group discovered that upon translation the heme-deficient β1 subunit (apo-β1) associates with the major cytosolic chaperone Hsp90 [129,130], which promotes its maturation. The heme moiety is then provided by GAPDH, which binds mitochondrially derived heme and delivers it to the β1 subunit [131,132]. The association of the β1 subunit with Hsp90 and the ATP-ase activity of HSP90 are essential for the translocation of heme from GAPDH to the apo-β1 [133]. Only after the insertion of heme Hsp90 dissociates from the β1 subunit and allows the binding of the α1 subunit and formation of the functional heterodimer [134]. It should be noted that substitution of the heme-coordinating His105 residue of the β1 subunit by phenylalanine or cysteine completely abolishes the binding of heme to SGC [21,118,135]. Nevertheless, mutant α1β1 heterodimers lacking the heme moiety fully retain the cGMP-forming activity and the ability to form a high-cGMP output state induced by protoporphyrin IX [21,118]. These studies strongly indicate that the insertion of heme is not an obligatory step in the assembly of a functional SGC heterodimer. Interestingly, NO also has a role in the maturation of SGC heterodimer. It is reported that physiological levels of NO promote the deployment of cellular heme and GAPDH-mediated heme insertion [136], demonstrating another role of NO in the function of SGC unrelated to the activation of cGMP-forming activity.

### 3.4. Proteins Affecting Sub-Cellular Localization of SGC

Although SGC is predominantly found in cytosolic fraction, a significant portion of the enzyme is membrane associated. Interaction of SGC with different proteins promotes this localization. For example, in the brain, 90% of the GC-2 isoform is found in the synaptic membrane. The interaction between the C-terminal region of the α2 subunits of GC-2 with the third PDZ domain of PSD-95 recruits GC-2 to the membrane fraction of synaptosomes [4]. In cardiomyocytes, the interaction of SGC with Hsp90 directs SGC to the plasma membrane of cardiomyocytes within the caveolae [137], where Caveolin 3 may also protect SGC heme from oxidation [138]. The translocation of SGC may also be facilitated by its interaction with AGAP1 and resulting phosphorylation [139]. In cardiomyocytes, the β1 subunit was shown to co-localize and co-precipitate with Connexin 43 at the intercalating discs [140]. The interaction of SGC and Connexin 43 was shown to be important in cardiac electrical function.

### 3.5. Interacting Proteins Altering the Response to NO

Application of different biochemical and genetic techniques identified a number of SGC interacting proteins that directly affect the activity SGC. The lysate of COS-7 cells, which do not express SGC, was reported to boost several fold the activity of resting and NO-activated SGC. Using a SGC affinity matrix Hsp70 was identified as a protein directly interacting with SGC [56]. The Hsp70/SGC complex was confirmed by immunoprecipitation and was shown to promote membrane localization of SGC. The immunodepletion of Hsp70 from the COS-7 cell lysate blocked the boosting of SGC activity by the lysate. The biological significance of this interaction remains to be unraveled.

By applying a yeast two-hybrid screening approach, the η subunit of the chaperonin containing t-complex polypeptide (CCTη) was identified as a protein interacting with the β1 subunit of SGC [141]. The N-terminal portion of the β1 subunit is crucial for this interaction. The complex between CCTη and SGC was confirmed by co-precipitation and cellular co-localization. The addition of purified CCTη to SGC preparation resulted in diminished activity of SGC stimulated by NO donors, but not by allosteric stimulator BAY41-2272 [141]. The functional role of such interaction is yet to be determined. However, considering the importance of CCT complex in the process of protein folding [142], it can be reasonably assumed that this interaction is involved in the maturation of SGC. As already mentioned, a similar inhibitory effect was described for the complex between oxidoreductase PDI and SGC [126]. In the same yeast two-hybrid screening, the G-protein-signaling modulator 2, also known as LGN for its 10 Leucine-Glycine-Asparagine repeats, was identified as a protein interacting with both α1 and β1 subunits of SGC [143]. The LGN/SGC complex was co-immunoprecipitated from cells expressing both proteins and from native tissues. This interaction required the N-terminal domain of LGN, but did not require the N-terminal portions of alpha1 or beta1 subunits. When overexpressed, LGN decreased the activity of cellular SGC, while the repression of LGN expression correlated with increased sGC activity. Interestingly, the inhibitory effect of LGN was observed only in the presence of cell lysates, indicating that additional cell components are needed. Additional studies are needed to determine the biological significance of this interaction.

### 3.6. Role of Protein Kinases in SGC Activity

It has been reported that cGMP generated by activated SGC modulates SGC activity via the engagement of cGMP-dependent phosphorylation. Activation of PKG in gastric smooth muscle cells in response to sodium nitroprusside-elicited elevation of cGMP results in phosphorylation and inhibition of SGC [144,145]. The results imply a feedback inhibition of soluble GC activity by PKG-dependent phosphorylation which impedes further synthesis of cGMP. Later studies performed with purified proteins confirmed partial inhibition of SGC by PKG. Mechanistic studies revealed that PKG directly phosphorylates the Ser64 residue of the α1 subunit, resulting in lower V_max_ and a blunted improvement of K_M_(GTP) in response to NO [146]. A different study reported that PKG-dependent inhibition of SGC is caused by the phosphorylation of the β1 subunit [147].

*In vitro* studies performed with purified SGC and cAMP-dependent kinase (PKA) demonstrated that phosphorylation of the α1 subunit increased SGC activity [148]. Later studies in GH(3) immortalized pituitary cells demonstrated that activation of PKA also enhanced the activity of cellular SGC [149]. Moreover, the expression of a constitutively active PKA in these cells resulted in enhanced SGC-dependent cGMP synthesis and phosphorylation of the α1 subunit. Modulation of SGC activity by PKG and PKA suggest that cAMP and cGMP nucleotides should also be regarded as important small molecule regulators of SGC.

Studies with SGC isolated from rat brains revealed that protein kinase C directly phosphorylates SGC, while Ca^2+^ and phorbol ester enhanced the level of SGC phosphorylation. cGMP-forming activity of SGC was potentiated by this PKC-dependent phosphorylation [150]. Considering that elevated cellular Ca^2+^ stimulated the translocation of SGC to cellular membrane [3], where SGC exhibited higher sensitivity to NO, it is possible that this process is mediated by Ca^2+^-dependent PKC.

## 4. Conclusions

The proper function of NO/SGC signaling is essential in the maintenance of multiple physiological functions. It is now clear that a plethora of cell- and tissue-derived small molecules and proteins modulates the activity of SGC (Table 1). Although most of these effects are well characterized *in vitro* on purified homogeneous systems or in specifically designed cellular models, it is important to further evaluate the contribution of these modulating cell factors on physiological function of SGC in vivo. SGC is an important therapeutic target. A growing number of SGC-targeting synthetic small molecules are being identified and introduced in clinics or tested in clinical trials. Cellular factors modulating the activity of SGC constitute an opportunity for improving the effectiveness of existing SGC-directed drugs and/or the creation of new therapeutic strategies.

## Figures and Tables

**Table 1:** Cellular factors modulating the activity and function of SGC.

Cellular Factor	Effect	References
**Role of additional NO as an allosteric factor**		
*Binding of additional NO to the proximal site of heme;* *Formation nitrosothiol or thionitroxide by additional NO* *Nitrosylation of sGC cysteines*	Stabilizes of NO:SGC adduct; enhances SGC activity; Enhances SGC activity Desensitizes of SGC towards NO	[66] [70] [78,79,81,83,84,85]
**Cell- and tissue-derived small molecules**		
*Free cellular thiols*	Reduction of oxidized SGC heme; Protects and reverses desensitization by nitrosothiols; Protects from inhibitory SGC thiol oxidation;	[27,102] [82] [87,88,89]
*Hydrogen sulfide*	Reduces oxidized SGC heme;	[104]
*Ca^2+^ ion*	Inhibits SGC via binding to two Ca^2+^-binding sites; Promotes translocation of SGC to membrane fraction.	[106,107,108] [3]
*Protoporphyrin IX*	Activates heme-deficient SGC	[117,118]
*Biliverdin IX*	Inhibits SGC activity	[119]
*Carnosine*	Inhibits SGC activation by NO	[122,123]
*Cobinamide*	Stimulates SGC activity	[114]
**Cellular proteins**		
*Protein targeting SGC thiols* -protein disulfide isomerase (PDI) -thioredoxin-1	Inhibits SGC activity Reverses S-nitrosylation of SGC	[126] [127]
*Protein affecting SGC heme* -CytB5R3 -Caveolin 3	Maintains SGC heme in ferrous state Possibly protects SGC heme in cardiomyocytes	[138]
*Proteins affecting SGC maturation* -Hsp90 -GAPDH	Promotes maturation of the β1 subunit; prevents premature binding of α1 Delivers heme to the β1 subunit	[129,130] [131,132,133]
*Protein affecting cellular localization* -PSD95 -Hsp90 -Connexin 43 -AGAP1	Localizes GC-2 to synaptosomes Directs SGC to caveolae in cardiomyocytes Binds SGC at the intercalating discs, affects cardiac electrical function Promotes SGC phosphorylation	[4] [137] [140] [139]
*Proteins affecting SGC activity* -CCTη -Hsp70 -LGN	Inhibits SGC activity upon binding Enhances SGC activity; promotes membrane localization Inhibits SGC in concert with unknown cellular factors	[141] [56] [143]
*Protein kinases affecting SGC* -PKG -PKA -PKC	Inhibition of SGC activity Stimulation of SGC activity Stimulation of SGC activity	[144,145,146,147] [148,149] [150]
